# FAM46C Expression Sensitizes Multiple Myeloma Cells to PF-543-Induced Cytotoxicity

**DOI:** 10.3390/biom15050623

**Published:** 2025-04-26

**Authors:** Annarita Miluzio, Federica De Grossi, Marilena Mancino, Stefano Biffo, Nicola Manfrini

**Affiliations:** 1 INGM, Istituto Nazionale Genetica Molecolare Romeo ed Enrica Invernizzi, 20122 Milan, Italy; 2 Department of Biosciences, University of Milan, 20133 Milan, Italy

**Keywords:** TENT5C, sphingosine kinase, PF-543, myeloma, cancer therapy

## Abstract

FAM46C is a tumor suppressor initially identified in multiple myeloma (MM) but increasingly recognized for its role also in other cancers. Despite its significance, studies exploring the therapeutic potential of FAM46C in combination with targeted treatments remain limited. Sphingosine kinases (SphK1 and SphK2) are key regulators of sphingolipid signaling, a pathway essential for maintaining cell structure and function but frequently deregulated in tumors, making them promising targets for cancer therapy. Preliminary work from our laboratory showed that FAM46C expression synergizes with administration of SKI-I, a pan-inhibitor of sphingosine kinases. In this study, we focused specifically on SphK1, the sphingosine kinase predominantly implicated in cancer and investigated the combinatorial effect of forced FAM46C expression and treatment with PF-543, a selective SphK1 inhibitor. We found that FAM46C overexpression enhances, whereas its downregulation reduces, the cytotoxic efficacy of PF-543 in MM cell lines. Using an in vivo xenograft model, we further validated these findings, showing that FAM46C-expressing MM tumors are indeed sensitive to PF-543 while tumors harboring the D90G loss-of-function variant of FAM46C are not. Overall, our results uncover a novel synergistic interaction between FAM46C expression and SphK1 inhibition, highlighting a promising therapeutic strategy for MM treatment.

## 1. Introduction

Multiple myeloma (MM) is a hematological malignancy characterized by the uncontrolled proliferation of plasma cells within the bone marrow, which, by accumulating, can lead to organ damage, including bone lesions, kidney dysfunction, anemia, hypercalcemia and bone marrow failure [1]. MM accounts for approximately 10% of all hematologic cancers and, despite advances in treatment, remains largely incurable due to disease relapse and the development of resistance to therapy [2,3]. Therefore, implementing novel therapeutic strategies based on novel targets becomes critical for improving long-term outcomes for patients.

FAM46C is a tumor suppressor protein originally characterized in MM [4,5,6,7], but now being recognized for its broader pan-cancer activity [8,9]. Beyond its role in cancer, FAM46C is also involved in other pathological contexts [10] and in physiological environments [11]. Although its mode of functioning is still widely debated [8], FAM46C has been proposed to function as either a non-canonical poly(A) polymerase [12,13] that stabilizes specific transcripts [4,14,15], an inhibitor of Polo-Like Kinase 4 (PLK4) that prevents centriole overduplication [16], or a master regulator of intracellular trafficking dynamics that indirectly inhibits autophagy [6]. Despite this mechanistic ambiguity, FAM46C pro-cell death features are well established and are starting to be considered for fine-tuning therapeutic strategies [8,17].

The sphingolipid metabolic pathway is a complex biochemical pathway responsible for the synthesis, modification, and degradation of sphingolipids, key components of cell membranes involved in signaling, apoptosis, and immune responses [18].

Sphingosine Kinases (SphKs) are central regulators in this pathway and are responsible for catalyzing the phosphorylation of sphingosines to produce sphingosine-1-phosphate (S1P), a bioactive lipid involved in cell proliferation, survival, and migration and fundamental for regulating catabolic lipid metabolism [19]. There are two known SphK isoforms: Sphk1 and Sphk2. SphK1 is more prominently implicated in cancer progression and chemoresistance [20], while SphK2, although involved in cell death and survival pathways, appears to function differently depending on the cellular context. Due to its strong association with tumor progression, selective targeting of SphK1 is emerging as a promising strategy in cancer therapy [19], and, recently, inhibition of SphK1 using selective agents, such as PF-543, has demonstrated promising cytotoxic effects in preclinical models by disrupting S1P signaling and sensitizing cancer cells to conventional therapies [19,21,22].

Previous studies from our lab have shown that functional FAM46C expression synergizes with SKI-I [6], a dual inhibitor of SphK1 and SphK2 [23,24,25]. In this study, we investigate the synergistic effects of FAM46C expression with the selective SphK1 inhibitor PF-543 both in vitro, in MM cell lines, and in vivo, in a mouse xenograft model.

## 2. Materials and Methods

### 2.1. Datamining of the DepMap Database

Analyses were performed using the DepMap Portal (https://depmap.org/portal/ (accessed on 10 January 2025); DepMap Public 24Q4 release) [26] as follows:

- At first, using the Celligner feature, we retrieved the full list of Plasma Cell Myeloma cell line models.
- Next, using the Data Explorer tool, we plotted PF-543, SKI-II and Fingolimod sensitivity against the mean gene effect of FAM46C, first, without clustering and then, with sub-clustering based on tumor type (primary, metastatic and other).
- Linear regression was then performed to assess the correlation between PF-543, SKI-II and Fingolimod sensitivity and the mean gene effect of FAM46C on: (1) the entire population or (2) each sub-cluster.
- Lastly, within each sub-cluster, only cell lines with a positive mean gene effect of FAM46C were analyzed exclusively for PF-543 drug sensitivity.

### 2.2. Cell Lines and Culturing

All the cell lines used in this study were purchased from the American Type Culture Collection (ATCC, Manassas, VA, USA). Testing for Mycoplasma contamination was performed every two weeks through PCR analysis.

LP1 cells were cultured in Iscove’s Modified Dulbecco’s Medium (IMDM, cat. No. ECB2072L, Euroclone, Pero, Italy), supplemented with 20% FBS (cat. No. ECS5000L, Euroclone), 100 U/mL penicillin, 100 mg/mL streptomycin (cat. No. ECB3001D, Euroclone) and 1% glutamine (cat. No. ECB3000D, Euroclone). OPM2, RPMI-8226, U266 and KMS-11 cell lines were, instead, grown in RPMI 1640 medium (cat. No. ECM2001L, Euroclone), containing 100 U/mL penicillin and 100 mg/mL streptomycin, 10% FBS and 1% glutamine.

LP1 and OPM2 cell lines harboring inducible constructs of wt *FAM46C* and of the *D90G* mutant allele described in [6] were used for re-expression experiments and were induced by administration of 2 μg/mL doxycycline hydrochloride (cat. No., D3447, Sigma-Aldrich, Milano, Italy) for 3 days.

RPMI-8226 and U266 cell lines expressing either a scramble control or shRNAs targeting FAM46C were produced, as described in [6], using lentiviral constructs purchased from Sigma-Aldrich:

- Scramble: MISSION pLKO.1-puro Non-Target shRNA Control (cat. No. SHC016).
- Sh1FAM46C: (cat no. SHCLNG-NM_017709, Clone ID TRCN0000168095).
- Sh2FAM46C: (cat no. SHCLNG-NM_017709; Clone ID TRCN0000166958).

LP-1 cell lines harboring inducible constructs of wt FAM46C expressing either a scramble control or shRNAs targeting FNDC3A were produced as described in [6].

### 2.3. RNA Extraction and RT-qPCR Analysis

Total RNA was extracted using Trizol (cat. No. 15596018, Life Technologies, Monza, Italy) and the RNeasy Mini Kit (cat. No. 74106, Qiagen, Milano, Italy). One ug of RNA was reverse transcribed using random primers and the SuperScript III First-Strand Synthesis SuperMix (cat. No. 18080-400, Life Technologies). The synthesized cDNA was then analyzed through RT-qPCR on a StepOne Real-Time PCR system (cat. No 4376357, Life Technologies). FAM46C levels were determined using TaqMan Universal PCR Master Mix (cat. No. 4324018, Life Technologies) and the Hs01933465_s1 probe (cat. No. 4331182, Life Technologies). 18S rRNA was used as an internal standard (cat. No. 4333760F, Life Technologies). SESN2 and FNDC3A levels were determined using the following oligonucleotides using β-Actin as a normalizer:

*β-ACTIN*_FWD:AGAGCTACGAGCTGCCTGAC;
*β-ACTIN*_REV:CGTGGATGCCACAGGACT;
*FNDC3A*_FWD:CCCAAGAATATATTTTCACTACTCCAA;
*FNDC3A*_REV:TTCACAAATGTGATCATTTACTTTCTC;
*SESN2_FWD*:GCGAGATCAACAAGTTGCTGG;
*SESN2_REV*:ACAGCCAAACACGAAGGAGG.

### 2.4. Protein Extraction and Western Blotting

SDS-PAGE, followed by western blot, was performed on protein extracts derived from OPM2 and LP1 cells with doxycycline inducible constructs after three days of doxycycline administration with or without 20 h of PF-543 treatment. Cells were collected and lysed in Radio Immuno Precipitation Assay buffer (10 mM Tris-HCl [pH 7.4], 1% sodium deoxycholate, 1% Triton X-100, 0.1% SDS, 150 mM NaCl, and 1 mM EDTA [pH 8.0]) as described in [27].

FAM46C levels were assessed using α-FLAG antibodies (1:1000 dilution; Sigma-Aldrich, cat. no. F1804), LC3B levels were detected using α-LC3B antibodies (1:1000 dilution; cat. No. 2775, Cell Signaling Technology, Leiden, The Netherlands), and GAPDH levels were detected using α-GAPDH antibodies (1:1000 dilution; cat. no. 2118, Cell Signaling Technology). Western blot signals were detected as described in [28].

### 2.5. In Vitro Drug Sensitivity Experiments

LP1 and OPM2 cell lines harboring doxycycline-inducible constructs for either wt *FAM46C* or *D90G* mutant allele expression were plated in 24 multi-well plates at a concentration of 500,000 cells/mL in 1 mL of media and treated with doxycycline (2 μg/mL) for 48 h. Then, 10, 20, 40, or 80 μM (respectively, 0.4, 0.8, 1.6 and 3.2 μL of a 25 mM stock) of PF-543 (cat. No. PZ0234, Sigma-Aldrich) were administered to the samples for 20 h.

RPMI-8226 U266 and KMS-11 cell lines expressing either a scramble control or shRNAs targeting FAM46C were plated in 24 multi-well plates at a concentration of 500,000 cells/mL in 1 mL of media. Twenty-four hours after plating, cells were treated with 10, 20, 40, or 80 μM (respectively, 0.4, 0.8, 1.6 and 3.2 μL of a 25 mM stock) of PF-543 for twenty hours.

Cell viability was then assessed using the Trypan Blue Exclusion Assay (cat. No. 15250061, Life Technologies) using a bright field microscope.

### 2.6. Cell Cycle and Apoptosis Analysis

For cell cycle and apoptosis analysis, LP1 cells harboring doxycycline-inducible constructs for either wt *FAM46C* or *D90G* mutant allele expression, were treated with doxycycline (2 μg/mL) for 48 h. Then, 20 and 40 μM PF-543 (cat. no. PZ0234, Sigma-Aldrich) were administered for 20 h.

For cell cycle analysis, 1 million cells were washed in PBS and fixed for 30 min with cold 70% ethanol. After washing, cells were stained in PBS containing 50 µg/mL of propidium iodide (PI) and 0.1 mg/mL of RNase A (cat. No. RNASEA-RO, Sigma-Aldrich) at 37 °C for 30 min. The stained cells were analyzed by flow cytometry on a BD FACSCanto II machine and the percentage of cells in G0/G1, S and G2 phases was determined using the cell cycle tool on FlowJo. A minimum of 10,000 events for each sample was acquired. Analyses were performed with BD FACSDIVA, version 9.0 and FlowJo software, version 10.8.1.

For apoptosis analyses, 500,000 cells were double stained using annexin V/propidium iodide (PI) based Dead Cell Apoptosis Kit (cat No. V13241, Life Technologies). The kit discriminates between live (double negative), early apoptotic (single annexin V-positive), late apoptotic (double Annexin V- and PI-positive) and necrotic (single PI-positive) cells. A minimum of 10,000 events for each sample were acquired using a BD FACS CANTO II flow cytometer. Analyses were performed with the BD FACSDIVA and FlowJo softwares.

### 2.7. Mouse Xenograft Model and Ex Vivo Experiment

Male NOD scid gamma mice (JAX™ NSG^®^ Mice NOD, Charles River, Calco, Italy) were used for xenograft experiments. The mice were injected with 3 × 10^6^ OPM2 MM cells harboring either the wt *FAM46C* gene or the *D90G* mutant allele under the doxycycline-inducible promoter. The grouping of animals was randomized. Once tumors reached a size of 200 mm^3^ (at day 16), the mice were administered daily doses of doxycycline (2.5 mg/kg) to induce expression of either wt *FAM46C* or of the *D90G* mutant allele. The mice were either left untreated (treated with DMSO) or administered with PF-543 (10 mg/kg) for a total of 8 days. At day 8 post-doxycycline induction, the mice were sacrificed and analyzed. N = 10.

All mice were maintained in pathogen-free conditions and experiments were performed in accordance with the guidelines of the Ethics Committee of San Raffaele Scientific Institute, Milan, Italy, following experimental protocols fulfilling E.C. regulations that were reviewed by the Institutional Animal Care and Use Committee (IACUC n. 688, authorization n. 764/2015-PR, 30 July 2015).

### 2.8. Quantitation and Statistical Analysis

All quantifications are expressed as means ± SD. Statistical *p* values were calculated using either two-tailed *t* tests or single factor ANOVA. NS: *p* > 0.05; *: *p* < 0.05; **: *p* < 0.01; ***: *p* < 0.001; ****: *p* < 0.0001.

## 3. Results

### 3.1. PF-543 Sensitivity Is Associated with MM Cell Line Dependencies on FAM46C

Previous results from the lab revealed a strict correlation between the gene expression associated with wt FAM46C and that triggered by SphK inhibition [6]. Moreover, our data demonstrated that re-expression of a functional FAM46C in MM cells synergized with administration of SphK1/SphK2 inhibitor SKI-I [6], suggesting that targeting the SphK pathway in FAM46C-expressing cells might be a promising therapeutic strategy for MM treatment. Given that, among the two known SphK isoforms (i.e., SphK1 and SphK2), SphK1 is more prominently involved in cancer progression while SphK2 has less defined and context-dependent effects [20], we decided to test the efficacy of specifically targeting Sphk1 in MM cell lines harboring different levels of FAM46C.

To do so, we selected the PF-543 compound due to its high potency and selectivity compared to other Sphk1 inhibitors [29,30].

**Figure 1:**
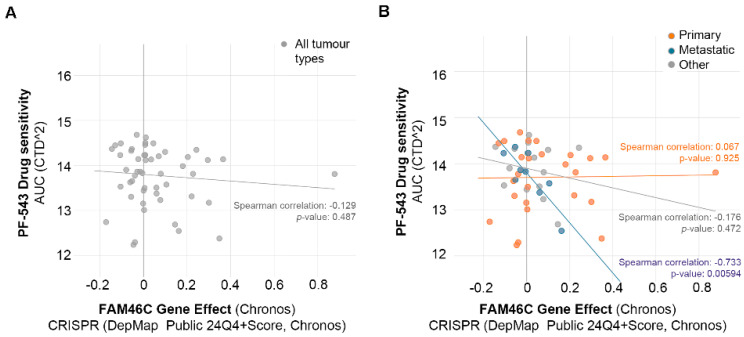
Sensitivity of MM cell lines to PF-543 treatment is associated with an increased FAM46C “gene effect” and with disease severity. (**A**) Scatter blot representing, for each MM cell line considered, the sensitivity to PF-543 treatment and the relative FAM46C gene effect. (**B**) Scatter blot representing the same data as in (**A**), but with cell lines grouped by tumor type. The Spearman correlation between the two ranked variables and the relative *p*-values are shown. The data were retrieved from, and images were produced with, DepMap, 24Q4 release.

To first assess the potential role of PF-543 in inducing cell death in MM cells, we explored the DepMap database (https://depmap.org/portal/ (accessed on 10 January 2025)) [26]. Taking advantage of the Cellligner integrated feature [31], we generated a list of plasma cell myeloma cell line models to analyze. Given that FAM46C functions as a tumor suppressor gene, we then explored if there was a correlation between cell line sensitivity to PF-543 and the anti-tumoral effect of FAM46C (Figure 1A,B).

To do so, we took advantage of the “gene effect” metric available in DepMap. The “gene effect” metric quantifies the impact of knocking out a specific gene on cancer cell line viability. Positive values indicate that the knockout enhances cell viability, suggesting a tumor suppressive function, while negative values indicate the opposite effect. When analyzing all tumor cell lines together, we observed a trend, albeit not statistically significant, suggesting correlation between PF-543 sensitivity and FAM46C gene effect (Figure 1A). However, correlation became highly significant when cell lines were subdivided by tumor type (primary or metastatic). Specifically, despite no significant correlation was found in primary tumor-derived cell lines, a significant correlation was found in metastasis-derived cell lines (Figure 1B), suggesting that increased sensitivity to PF-543 is associated not only with FAM46C gene effect, but also with tumor aggressiveness. Further supporting this result, when we focused only on cell lines with a positive FAM46C gene effect, metastasis-derived cell lines were the most sensitive to PF-543 (Figure S1).

These preliminary findings suggested a strong association between FAM46C expression, its tumor-suppressor function, and PF-543 sensitivity.

### 3.2. Upregulation of FAM46C in MM Cells Synergizes with PF-543 Treatment

**Figure 2:**
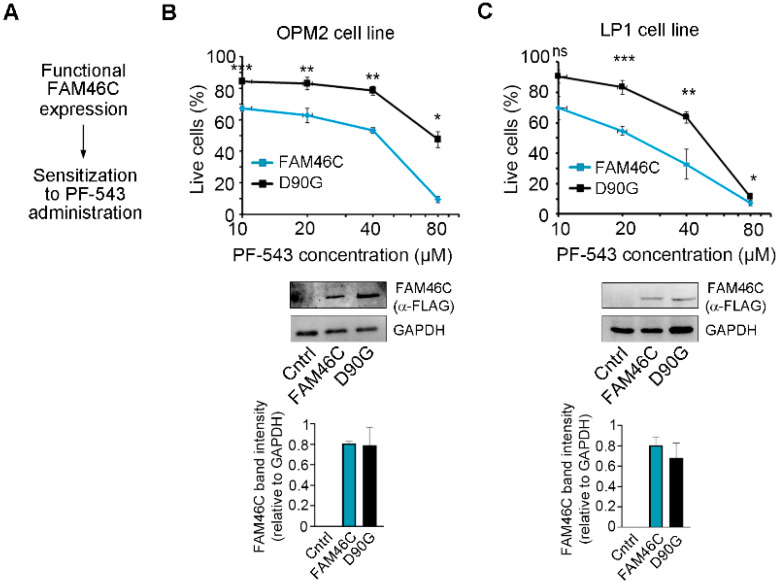
Expression of a functional FAM46C sensitizes MM cells to PF-543 treatment. (**A**) Schematic representation of our driving hypothesis. (**B**) Top, drug sensitivity curves of MM OPM2 cells expressing either wt *FAM46C-FLAG* (FAM46C) or the *D90G-FLAG* mutant allele (D90G). After 48 h of doxycycline induction, cells were treated for 20 h with increasing doses of PF-543. Center, Western blot showing wt FAM46C-FLAG or D90G-FLAG protein levels in OPM2 cell lines. GAPDH was used as loading control. Control: parental OPM2 cell line. Bottom, histograms representing FAM46C-FLAG band intensities normalized on GAPDH signals. (**C**) Same as in (**B**), except that experiments were performed in LP-1 MM cells. Control: parental LP1 cell line. Values in drug sensitivity curves and in band intensity histograms represent the mean ± SD of three independent experiments. Statistical *p*-values were calculated using two-tailed *t* tests, ns: *p*-value > 0.05; *: *p*-value < 0.05; **: *p*-value < 0.01; ***: *p*-value < 0.001.

Having established a correlation between the *FAM46C* gene effect and cell line sensitivity to PF-543, we next sought to validate these results in vitro using MM cell lines. At first, we tested if re-expression of wt FAM46C in MM cell lines lacking a functional FAM46C could render them more sensitive to PF-543 treatment (Figure 2A). To do so, we used two different cell lines: LP-1 cells, which harbor deletion of both *FAM46C* alleles, and OPM2 cells, harboring deletion of one *FAM46C* allele and mutation of the other [6]. We re-expressed wt FAM46C using our previously described doxycycline-inducible construct [6] and treated cells with increasing doses of PF-543. To account for potential dominant-negative or compensatory effects, we used expression of the well-established *D90G* loss-of-function variant of *FAM46C* as a control instead of an empty vector. This ensured that any observed phenotypic differences were specifically due to the functional activity of wt FAM46C rather than nonspecific effects of gene overexpression. We found that wt *FAM46C* expression significantly sensitized both LP1 and OPM2 cells to PF-543 administration compared to expression of the *D90G* loss-of-function variant (Figure 2B,C, western blot original images can be found in Supplementary Figure S2). These results suggest that reconstitution of a functional FAM46C can actually sensitize MM cells to PF-543 administration.

### 3.3. Downmodulation of FAM46C in MM Cells Antagonizes PF-543 Treatment

**Figure 3:**
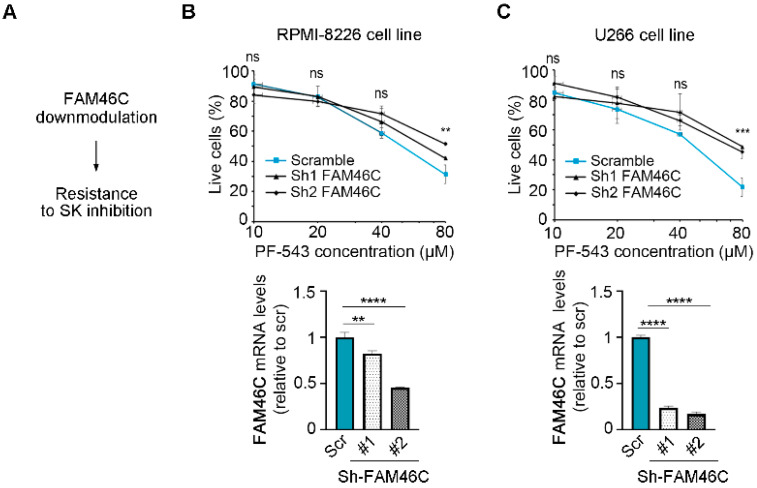
Downmodulation of functional FAM46C desensitizes MM cells to PF-543 administration. (**A**) Schematic representation of our driving hypothesis. (**B**) Top, drug sensitivity curves of MM RPMI-8226 cells with FAM46C downmodulation. 24 h after plating, cells were treated for 20 h with increasing doses of PF-543. Bottom, RT-qPCR showing the levels of endogenous FAM46C relative to 18S. Scr: scramble control; #1: FAM46C sh1, #2: FAM46C sh2. (**C**) Same as in (**B**), except that experiments were performed in U266 MM cells. Values represent the mean ± SD of three independent experiments. Statistical *p*-values were calculated using single factor ANOVA for drug sensitivity experiments and two-tailed *t* tests for RT-qPCR data. ns: *p*-value > 0.05; **: *p*-value < 0.01; ***: *p*-value < 0.001; ****: *p*-value < 0.0001.

Next, we wanted to confirm these results through a different experimental approach, specifically by testing if downmodulation of FAM46C in MM cells expressing high levels of functional FAM46C could indeed render cells more resistant to PF-543 treatment (Figure 3A). We used RPMI-8226, U266, and KMS-11 cell lines, which harbor high levels of functional FAM46C [6], and downmodulated endogenous FAM46C using two different shRNAs. In all the cell lines analyzed, the two different shRNAs downmodulated FAM46C mRNA levels to different extents (Figure 3B(bottom), Figure 3C(bottom) and Figure S3(bottom)). Despite the different knockdown efficiency, all cell lines with reduced FAM46C levels exhibited increased resistance to PF-543 treatment compared to control cells (Figure 3B(top), Figure 3C(top) and Figure S3(top)), indicating that a decreased amount of functional FAM46C is actually associated with decreased responsiveness to PF-543 treatment, confirming our previous results.

Altogether, the data obtained indicate that expression of a functional FAM46C is capable to sensitize MM cell lines to PF-543 treatment.

### 3.4. Upregulation of FAM46C in a MM Xenograft Model Sensitizes Cells to PF-543 Administration

**Figure 4:**
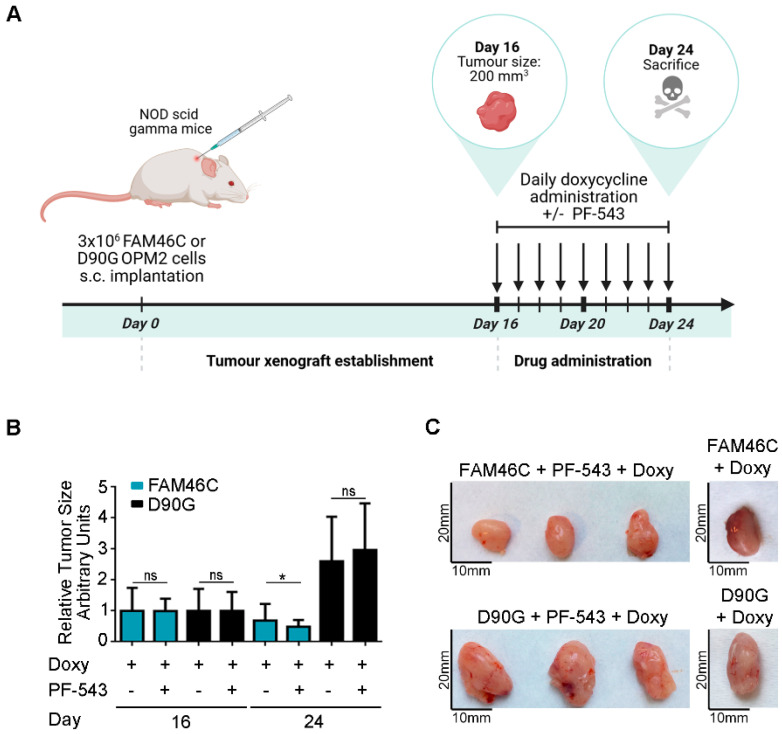
Expression of a functional FAM46C sensitizes MM tumors to PF-543 administration in an in vivo xenograft mice model. (**A**) Schematic representation of our in vivo experimental approach. NOD scid gamma mice were injected with 3 × 10^6^ OPM2 MM cells harboring either wt FAM46C or D90G mutant doxycycline-inducible constructs. Once tumors reached a size of 200 mm^3^ (day 16) mice were administered daily doses of doxycycline (2.5 mg/kg) and were, either left untreated, or treated with daily doses of SphK1 inhibitor PF-543 (10 mg/kg) for 8 days. The mice were then sacrificed (at day 24). Tumor sizes were monitored daily. The image was created with BioRender, https://BioRender.com, accessed on 16 April 2025 (**B**) Effects of PF-543 administration on tumor growth in vivo. Histograms represent mean tumor volumes ± SD of 10 independent tumors, calculated at the days indicated. Tumor volume measurements were performed using a caliper. (**C**) Representative images of tumors either treated or not with PF-543. Statistical *p*-values were calculated using two-tailed *t* tests, ns: *p*-value > 0.05; *: *p*-value < 0.05.

To further validate our in vitro findings in a more physiologically relevant setting, we conducted in vivo experiments using NOD scid gamma mice. The MM xenograft models were established with the OPM2 human MM cell line, harboring either wt *FAM46C* or *D90G* mutant doxycycline-inducible constructs. To assess the therapeutic effects of SPHK1 inhibition, mice were treated with PF-543 for eight days, with concomitant doxycycline administration (Figure 4A).

As expected, tumors derived from cells expressing wt *FAM46C* were smaller compared to those expressing the *D90G* mutant [6] (Figure 4B,C(right)). Strikingly, only tumors expressing wt *FAM46C* responded to PF-543 treatment, exhibiting a significant reduction in size after eight days of drug administration, whereas *D90G*-expressing tumors remained unaffected (Figure 4B,C).

These results indicate that expression of a functional FAM46C enhances sensitivity to PF-543 also in an ex vivo model of MM, with only FAM46C-expressing tumors responding to the drug at the administered dose.

Overall, our data indicate that expression of a functional FAM46C synergizes with administration of SphK1 inhibitor PF-543 to promote MM cell death.

### 3.5. FAM46C Synergizes with PF-543 Treatment by Triggering Apoptosis and Enhancing the UPR

Next, we aimed to define the specific mechanism(s) underlying the synergism between FAM46C expression and PF-543 treatment.

Previously, our lab had demonstrated that, in MM cells, FAM46C functions as a tumor suppressor by slowing down cell cycle progression and triggering apoptosis. These downstream phenotypes are associated with FAM46C-dependent hyperactivation of the Unfolder Protein Response (UPR) via ATF6 signaling and with autophagic dampening [6]. Therefore, we sought to determine which of these phenotypes contributed to the observed sensitization to PF-543.

**Figure 5:**
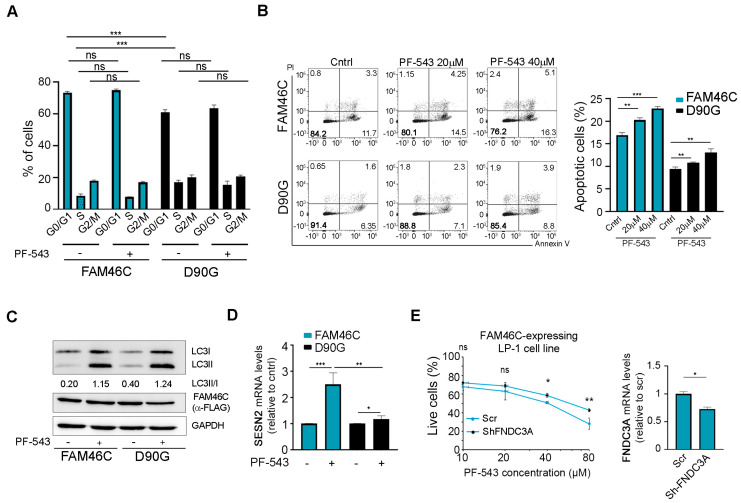
Expression of a functional FAM46C synergizes with PF-543 treatment by enhancing apoptosis and hyperactivating the UPR. (**A**) Cell cycle progression, (**B**) apoptotic rates, (**C**) accumulation of lipidated LC3B, and (**D**) SESN2 levels of LP-1 MM cells expressing either wt *FAM46C* or the *D90G* mutant allele either in the presence or absence of PF-543. After 48 h of doxycycline induction, LP1-MM cells expressing either wt *FAM46C* or the *D90G* mutant allele were treated for 20 h with the indicated doses of PF-543. (**A**) Cell cycle progression was then assessed through PI staining, (**B**) apoptosis induction through Annexin V/PI staining, (**C**) accumulation of lipidated LC3B through western blot, and (**D**) SESN2 levels through RT-qPCR with β-Actin normalization. Unless otherwise indicated, 20 µM of PF-543 was used. (**E**) Left, drug sensitivity curves of MM LP-1 cells expressing wt FAM46C with or without FNDC3A downmodulation. After 48 h of doxycycline induction LP-1 MM cells expressing FAM46C and either a scr control or ShRNAs targeting FNDC3A, were treated for 20 h with increasing doses of PF-543. Right, RT-qPCR showing the levels of endogenous FNDC3A relative to β-Actin. Scr: scramble control. Values represent the mean ± SD of three independent experiments. Statistical *p*-values were calculated using two-tailed *t* tests. ns: *p*-value > 0.05; *: *p*-value < 0.05; **: *p*-value < 0.01; ***: *p*-value < 0.001.

At first, we checked if FAM46C expression could affect cell cycle progression upon PF-543 treatment. As shown in Figure 5A, FAM46C expression alone led to an accumulation of cells in the G0 phase and a reduction in cells in the S phase, as previously reported [6]. However, PF-543 treatment did not significantly alter cell cycle progression in either FAM46C- or D90G-expressing cells, suggesting that the FAM46C/PF-543 synergism is not linked to cell cycle modulation.

We then focused on programmed cell death and tested if FAM46C expression affected apoptosis induction in PF-543-treated cells.

As expected, untreated FAM46C-expressing cells exhibited significantly higher apoptosis levels compared to D90G-expressing cells (Figure 5B). This apoptotic effect was further enhanced when cells were treated with PF-543, suggesting that FAM46C synergizes with PF-543 administration primarily through apoptosis induction.

Given that FAM46C-dependent apoptosis induction is associated with FAM46C indirect inhibition of autophagy [6] and that PF-543 was shown to induce autophagy [32,33], we next assessed if upon PF-543 treatment in the presence of wt FAM46C there was a significant alteration of the autophagic flux. As expected, untreated cells expressing FAM46C accumulated less lipidated LC3B (Figure 5C; western blot original images can be found in Supplementary Figure S4), a marker of autophagosome formation, compared to D90G-expressing cells. However, PF-543 treatment induced autophagy to a similar extent in both FAM46C- and D90G-expressing cells, suggesting that FAM46C-dependent dampening of autophagy is overridden by PF-543. These results suggest that FAM46C does not synergize with PF-543 treatment via autophagic modulation.

Since FAM46C was shown to trigger the UPR downstream of ATF6 signaling [6], we tested if hyperactivation of this pathway could contribute to the observed sensitization to PF-543. Upon PF-543 treatment, FAM46C-expressing cells strongly upregulated the mRNA levels of SESN2 (Figure 5D), a known downstream target of ATF6 [34], while D90G-expressing cells did not, suggesting that FAM46C may sensitize cells to PF-543 treatment, at least in part, through on UPR hyperactivation.

Given that FAM46C functions by localizing at the ER thanks to interaction with the FNDC3A protein, which is required for all FAM46C-induced phenotypes [6,7], we tested if the presence of FNDC3A was required for FAM46C-induced cytotoxicity upon PF-543 treatment. This seems indeed to be the case, as downmodulation of FNDC3A in FAM46C-expressing LP-1 cells rendered cells less sensitive to PF-543 administration (Figure 5E).

Overall, our results demonstrate that FAM46C expression synergizes with PF-543 treatment primarily by enhancing apoptosis and, possibly, by modulating UPR signaling downstream of ATF6.

## 4. Discussion

**Figure 6:**
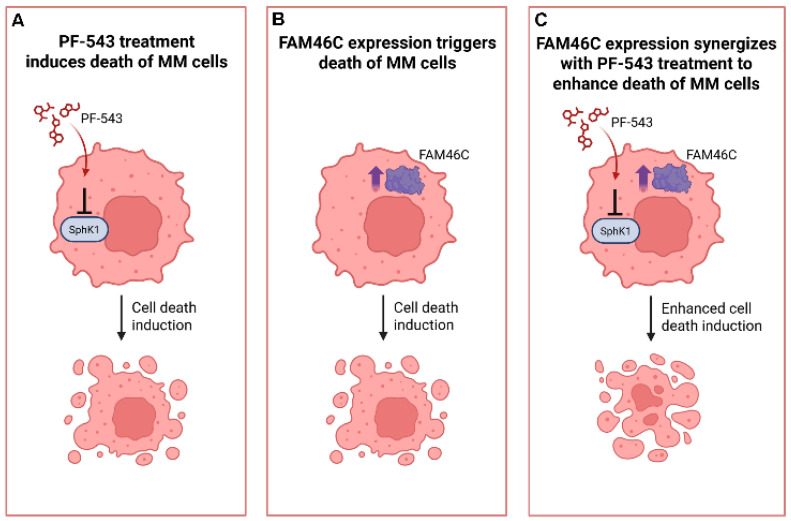
Expression of a functional FAM46C synergizes with PF-543 treatment to induce MM cell death. Cartoon summarizing our findings. (**A**) SPhk1 I inhibition through PF-543 administration induces cell death in MM cells. (**B**) Expression of functional FAM46C induces cell death in MM cells. (**C**) wt FAM46C expression synergizes with PF-543 administration to induce death of MM cells. The image was created with BioRender, https://BioRender.com, accessed on 16 April 2025.

Our study demonstrates that FAM46C expression enhances the sensitivity of MM cells to the SphK1 inhibitor PF-543, both in vitro and in vivo (Figure 6). We found that reintroducing a functional *FAM46C* allele in MM cells increased their susceptibility to PF-543, whereas downmodulating *FAM46C* reduced drug sensitivity. Furthermore, our MM xenograft model confirmed these results, as only tumors expressing wild-type *FAM46C* responded to PF-543 treatment. These findings suggest that FAM46C plays a crucial role in modulating MM cell response to SphK1 inhibition and highlight FAM46C expression as a potential biomarker for therapeutic response.

Beyond PF-543, several other SphK1 inhibitors are currently under investigation [19,35]. Future studies should determine whether wild-type FAM46C expression synergizes with these alternative SphK1 inhibitors or if the observed effect is specific to PF-543.

Since we have previously demonstrated that FAM46C also synergizes with the SphK pan-inhibitor SKI-I [6], we hypothesized that this effect may be generalizable across SphK1 inhibitors. However, when we explored the DepMap portal for other inhibitors of the SphK pathway, namely SKI-II, a dual SphK1 and SphK2 inhibitor [36,37], and Fingolimod, a nonselective functional antagonist of S1P that also inhibits SphK1 [38,39], we found no association with FAM46C gene effect (Figure S5A–D), indicating that the situation might be more complex than expected and would require a detailed case-to-case validation.

Several studies have indicated that the highest efficacy of SphK1 inhibitors is achieved when combined with other antitumoral drugs [19]. For example, PF-543 has been shown to synergizes with 5-FU and doxorubicin [40], while Safingol [41] synergizes with synthetic flavonoid 2-nitroflavone (2′-NF), enhancing breast cancer cell death [42]. On this line, it would be tempting to speculate that FAM46C might also synergize with such combination treatments. Future experiments should further investigate this possibility.

At the molecular level, the precise mechanism of action of FAM46C remains a subject of debate, with three major models attempting to explain its function [8]. The model proposed by our lab suggests that FAM46C functions at the intersection of intracellular trafficking and secretion [6]. Specifically, by modulating intracellular trafficking dynamics, FAM46C dampens autophagy, leading to an accumulation of protein aggregates and subsequent apoptosis. Based on this idea, we have recently proposed that FAM46C might sensitize cells differentially to autophagy modulators [17]. SphK1 inhibition was shown to trigger the autophagic pathway [32,33], bringing us to the question of whether FAM46C-induced sensitization to SphK1 inhibition was any way associated with the remodulation of autophagy triggered by FAM46C. Here, we found that PF-543 is capable of triggering autophagic induction to the same extent in both FAM46C- and D90G-expressing cells, apparently abrogating FAM46C inhibitory effect on autophagy. However, future studies must confirm these results and fully dissect the relevance of the FAM46C-SphK1 interaction for cancer therapy.

FAM46C was shown to function as a tumor suppressor in MM by interacting with ER-bound protein FNDC3A [6,7]. Given that FNDC3A is also mutated in a fraction of MM patients [6] and that its expression was shown to be associated with sensitivity to anti-cancer drugs in MM cell lines [17], here we tested if FNDC3A could have a role in FAM46C-induced sensitization to PF-543 administration. We found that this is indeed the case, as FNDC3A downmodulation reduces the sensitivity of FAM46C-expressing MM cells to PF-543 treatment. However, future studies must confirm this result and better define the extent of FNDC3A involvement in PF-543 sensitization.

Last but not least, given the increasing relevance of FAM46C in other cancer types, such as hepatocellular carcinoma, colorectal, and prostate cancer [8], future investigations should extend beyond MM. Dissecting the potential synergy between FAM46C expression and SphK1 inhibition in additional tumor models could reveal whether FAM46C functions through conserved mechanisms across different malignancies or if its tumor-suppressive role is context-dependent. Understanding these dependencies and defining at the molecular level if and how FAM46C is affecting sphingosine metabolism, could provide valuable insights into the broader applicability of targeting FAM46C-SphK1 interactions in cancer therapy.

Moreover, our results may ultimately contribute to implement therapy intervention, as stratifying patients based on functional FAM46C expression, may predict their sensitivity to SphK inhibitors.

## 5. Conclusions

In this study, we have demonstrated that expression of functional FAM46C in MM cells synergizes with the cytotoxic effects induced by SphK1 inhibitor PF-543. This synergism is specifically driven by FAM46C-induced apoptosis and, possibly, UPR activation downstream of ATF6.

## Data Availability

Data are contained within the article and supplementary materials.

## Supplementary Materials

The following supporting information can be downloaded at https://www.mdpi.com/article/10.3390/biom15050623/s1. Figure S1: Sensitivity to PF-543 administration increases with disease severity in MM cells with a positive FAM46C gene effect; Figure S2: Uncropped images related to western blots of Figure 2. Figure S3: Downmodulation of functional FAM46C desensitizes KMS-11 MM cells to PF-543 treatment. Figure S4: Uncropped images related to western blots of Figure 5. Figure S5: Sensitivity of MM cell lines to SKI-II or Fingolimod treatment is not associated with FAM46C “gene effect” nor with disease severity.
