# Supplementary material for: FAM46C Expression Sensitizes Multiple Myeloma Cells to PF-543-Induced Cytotoxicity
Source: Biomolecules. 2025 Apr 26;15(5):623. doi: 10.3390/biom15050623 (PMC12109155; doi:10.3390/biom15050623)
Supplement: Supplementary file 1 [file biomolecules-15-00623-s001.zip › biomolecules-3504907-supplementary.pdf]

Supplementary Figures

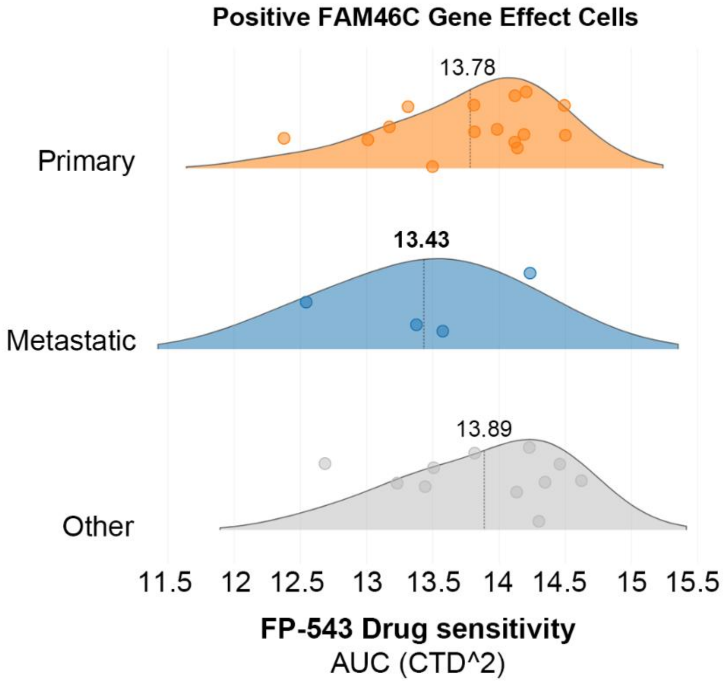

Figure S1. Sensitivity to PF-543 treatment increases with disease severity in MM cells with a positive FAM46C gene effect. 1D density plots on the same cells as in Figure 1 selected for positive values of FAM46C gene effect and grouped by tumour type. Data were retrieved from, and images were produced with DepMap, 24Q4 release.

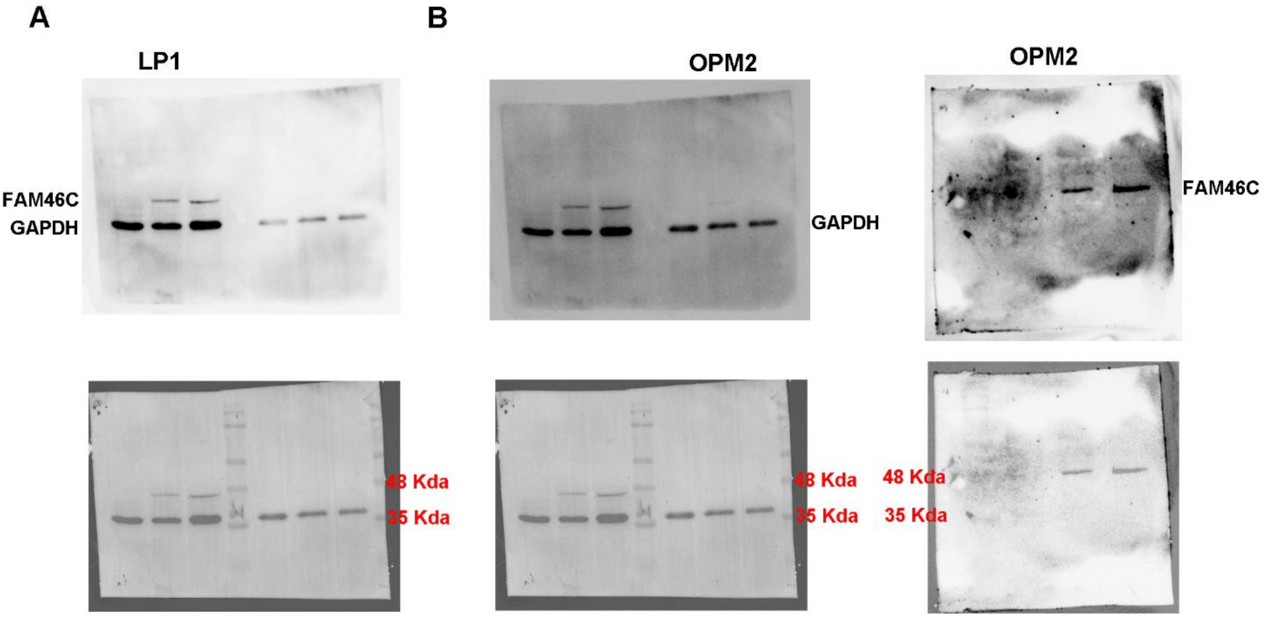

Figure S2. Uncropped images related to western blots of Figure 2. (A) Uncropped images related to panel C of Figure 2. (B) Uncropped images related to panel B of Figure 2.

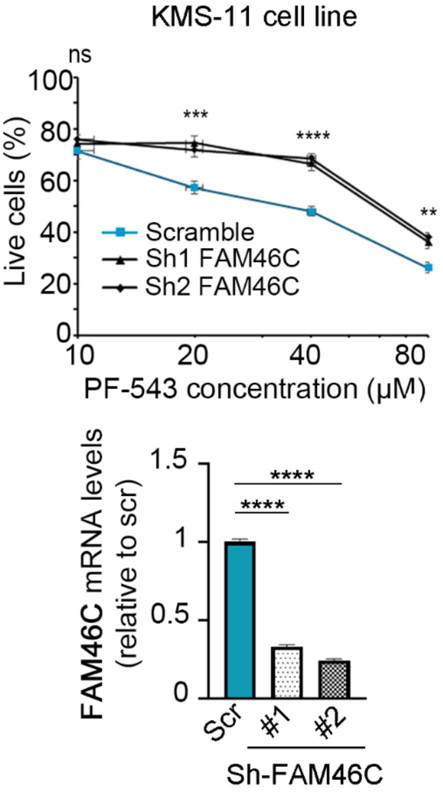

Figure S3. Downmodulation of functional FAM46C desensitizes KMS-11 MM cells to PF-543 treatment. Top, drug sensitivity curves of MM KMS-11 cells with FAM46C downmodulation. 24 hrs after plating, cells were treated for 20 hrs with increasing doses of PF-543. Bottom, RT-qPCR showing the levels of endogenous FAM46C relative to 18S. Scr: scramble control; #1: FAM46C sh1, #2: FAM46C sh2.

Values represent the mean +/- SD of three independent experiments. Statistical *P*-values were calculated using single factor ANOVA for drug sensitivity experiments and two-tailed t tests for RT-qPCR data. ns: *P*-value > 0.05; **: *P*-value < 0.01; ***: *P*-value < 0.001; ****: *P*-value < 0.0001.

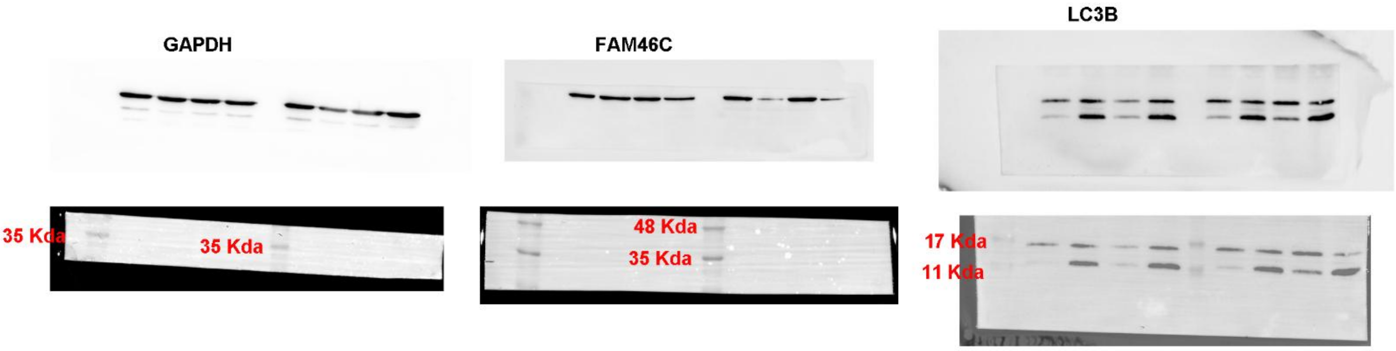

Figure S4. Uncropped images related to western blots of Figure 5. Uncropped images related to panel C of Figure 5.

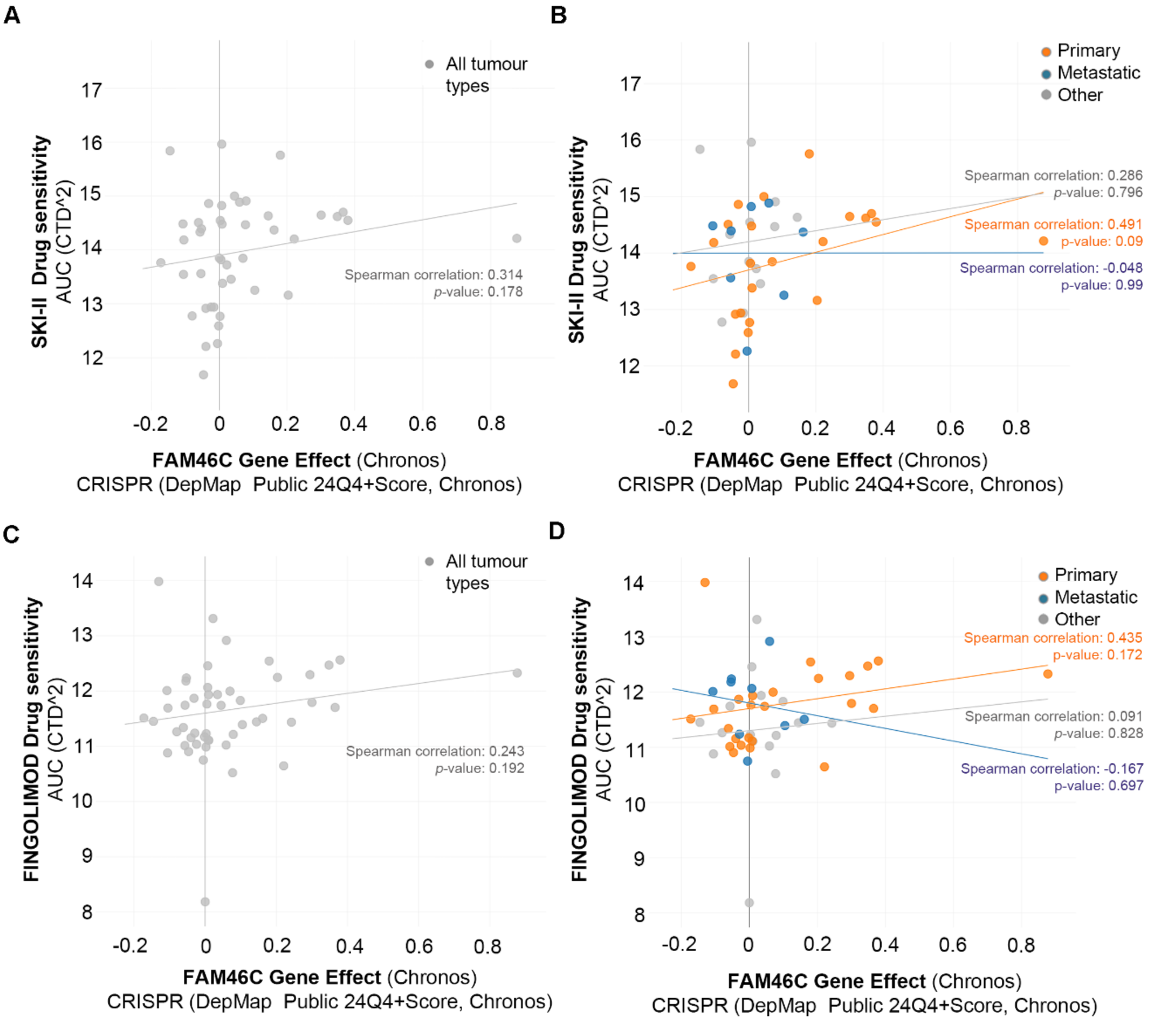

Figure S5. Sensitivity of MM cell lines to SKI-II or Fingolimod treatment is not associated with FAM46C "gene effect" nor with disease severity. (A,C) Scatter blots representing, for each MM cell line considered, the sensitivity to either SKI-II or Fingolimid administration and the relative FAM46C gene effect. (B, D) Scatter blots representing the same data as in A or C, but with cell lines grouped by tumor type.

Spearman correlation between the two ranked variables and the relative *P*-values are shown. Data were retrieved from, and images were produced with, DepMap, 24Q4 release.
